# Stakeholder Analysis of Community Distribution of Misoprostol in Lao PDR: A Qualitative Study

**DOI:** 10.1371/journal.pone.0162154

**Published:** 2016-09-15

**Authors:** Jo Durham, Melissa Warner, Alongkone Phengsavanh, Vanphanom Sychareun, Viengnakhone Vongxay, Keith Rickart

**Affiliations:** 1 Faculty of Medicine, School of Public Health, The University of Queensland, Brisbane, Queensland, Australia, 4006; 2 University of Health Sciences, Vientiane, Lao PDR; 3 Communicable Diseases Unit, Chief Health Officer Branch, Health Service & Clinical Innovation Division, Department of Health, Queensland Government, Brisbane, Queensland Australia, 4006; University of Liverpool, UNITED KINGDOM

## Abstract

**Background:**

Globally, significant progress has been made in reducing maternal mortality, yet in many low-resource contexts it remains unacceptably high. Many of these deaths are due to postpartum haemorrhage and are preventable with access to essential obstetric care. Where there are barriers to access, maternal deaths could be prevented if community-level misoprostol was available. The purpose of this study was to explore perceptions of stakeholders regarding misoprostol use in the Lao People’s Democratic Republic, a setting with high maternal mortality.

**Methods:**

Semi-structured interviews were conducted with 35 stakeholders in the capital, Vientiane and in one northern province identified as a site for a possible intervention. The sample included international and national stakeholders involved in policy-making and providing maternal and reproductive health services.

**Findings:**

Most stakeholders supported a pilot program for community distribution of misoprostol but levels of awareness of the drug’s use in preventing postpartum haemorrhage and level of influence over policy direction varied considerably. Some international organizations, all identified as powerful in influencing policy, were ambivalent about the use of community distribution of misoprostol. Concerns related to the capacity of village health workers or lay people to safely administer misoprostol, whether its distribution would undermine efforts to improve access to safe delivery services and active management of the third stage of labour, the ease with which prescription drugs can be bought over the counter, and technical, logistical, and financial constraints.

**Conclusion:**

Access to appropriate oxytocic drugs is a matter of health equity. In settings without access to essential obstetrical care, misoprostol represents a viable solution for the prevention of postpartum haemorrhage. Understanding stakeholders’ perspectives and their legitimate concerns on misoprostol can inform interventions in order to assuage these concerns and enable disadvantaged women to access misoprostol and its potentially life-saving benefits.

## Background

Significant progress has been made globally in reducing maternal mortality. Improvements however have not been uniform and in many parts of the world maternal mortality remains unacceptably high with 99 per cent of maternal deaths occurring in developing countries [1]. Disparities are also seen with maternal mortality higher in rural communities and poorer populations at a national level. [1]. Many of these deaths are due to postpartum haemorrhage (PPH) and could be prevented if women had access to a skilled birth attendant and essential obstetric care [1, 2]. Many women however, do not have access to such care and give birth at home attended by a traditional birth attendant (TBA), a relative, or on their own. Misoprostol, a generic, low cost, heat-stable oxytocic that can be administered in tablet form, has the potential to mitigate PPH and with training, can be safely administered by low-level health staff or even by women themselves [2–4]. Further, misoprostol for the prevention of PPH does not require a diagnosis of PPH as it can be given prophylactically to every woman immediately after delivery [5]. While universal access to health facilities, oxytocin, and skilled healthcare provider attendance at delivery, must be the goal in low-resource settings, the community distribution of misoprostol provides an achievable interim solution to substantially reducing the maternal mortality rate (MMR) as a result of PPH [2, 4, 6–9]. In light of the evidence in support of the community distribution of misoprostol, the International Federation of Gynecology and Obstetrics (FIGO) [10] and the World Health Organization (WHO) [11] have endorsed the approach of preventing PPH by the administration of misoprostol by community or lay healthcare workers where access to essential obstetric care is not available.

The Lao People’s Democratic Republic (PDR) is a small, mountainous, multi-ethnic country in Southeast Asia transitioning from a low to middle income country. It has a population of 6.5 million people, spread across 17 provinces [12]. Despite experiencing sustained economic growth over the last decade, it remains one of the least developed countries in Asia and is heavily dependent on foreign aid [13, 14]. While health indicators have improved, they remain amongst the worst in the region [15]. According to the Lao Social Indicator Survey (LSIS) 2011–12 [12], the MMR is estimated to be 357 per 100,000 live births [12], the highest in the region. In neighbouring Cambodia for example, the 2010 demographic health survey estimated the MMR to be 206 per 100,000 live births [16]. While attendance by a health professional at delivery in the Lao PDR increased dramatically in the five years prior to the LSIS, from 20 percent to 42 per cent, inequalities remain in healthcare access and maternal health outcomes. The proportion of urban women assisted at delivery by a health professional (80 per cent) for example, is more than double that of women in rural areas (31 per cent) [12]. Inequalities are also observed by ethnicity with over half of women in Lao-Tai (the main ethnic group) headed households assisted by a health professional at delivery, compared with only one in five women in other ethno-linguistic groups [12]. The delivery of healthcare services is organised through central hospitals (tertiary level), provincial hospitals (secondary level), district hospitals (the first level referral system), and health centres which provide healthcare at the village level. Maternal and child health services are provided throughout the country with outreach and in-facility services combined.

Improving access to a trained health professional is constrained by poverty, geography, poor road and transport infrastructure, a critical shortage of skilled birth attendants, and cultural, lifestyle and linguistic differences related to ethnicity [17–21]. Postpartum haemorrhage is the leading cause of maternal mortality in the Lao PDR [12]. While ultimately all pregnant women need accessible, appropriate and affordable essential obstetric care, in the meantime, the community distribution of misoprostol has been proposed as a viable option to reduce maternal mortality due to PPH. Despite the evidence in support of the use of the community distribution of misoprostol, there are reservations surrounding its use.

Resistance to the community distribution of misoprostol relates mainly to its possible use as an abortive agent. Abortion is legally restricted in the Lao PDR and due to concerns of possible misuse of misoprostol there remains debate over who should distribute and administer misoprostol safely in the community. Unregulated use in the community for termination of pregnancy or induction of labour can result in miscarriage, unintended early delivery and life threatening uterine rupture [5]. There is no evidence however, that inappropriate use of misoprostol by community workers or women themselves is a major problem [2, 4, 9]. Evidence from other low-resource settings suggests that pregnant women and others likely to be at the birth, are capable of using misoprostol safely, providing they have been provided with appropriate information, including precautions with each type of use, timing and dosages, management of side-effects, appropriate blood loss measurement techniques, and timely health facility referral and follow up if PPH continues or worsens [2, 4, 7, 9, 22, 23]. Considering the life-saving potential of misoprostol it is important to understand key stakeholders’ concerns and seek to address them in the development of a pilot program in the Lao PDR. Understanding stakeholder perspectives is vital not only for informing decision-makers and assisting the future design and implementation of effective and sustainable policies, but also for building consensus [24, 25]. In this paper, we present a stakeholder analysis undertaken to identify relevant actors, and understand their interests and influence on the maternal health policy-making process. The objective of the stakeholder analysis was to better understand the policy context to determine the acceptability and feasibility of introducing the distribution of misoprostol by community-based providers to women who give birth at home without a trained health professional in the Lao PDR. The stakeholder analysis enabled us to develop a communication plan for different stakeholder groups. This paper explains the process and findings of the stakeholder analysis and may be useful for other low-resource settings characterized by high home birth rates in remote village locations. It also shows how stakeholder analysis can be used to increase knowledge about stakeholder concerns around community distribution of misoprostol and also help to develop strategies to manage these concerns.

## Methods

This qualitative study followed a stakeholder analysis approach, seeking to gather knowledge about policy actors and their interests, degree of influence, and available resources [26, 27]. Stakeholders are actors that have direct impact on an issue or can affect it indirectly by enhancing or weakening the authority of key decision-makers and influencing implementation processes [26, 27]. For the purpose of this study, the scope was intentionally focused on community distribution of misoprostol. The timeframe was limited to the analysis of national policy-making dynamics at the time of the field research (2013). The primary sources of data for the stakeholder analysis were 35 semi-structured in-depth interviews.

Qualitative research was deemed the most appropriate study method as it enabled the researcher to gain an in-depth understanding and capture the different meanings and perspectives of stakeholders surrounding the use of misoprostol [28, 29]. Further, the intent was not to generalise beyond the site where the data were collected. While the overall design for this study was qualitative, the analysis included quantifying some data. Incorporating numbers in qualitative research can contribute to the internal generalisability of claims within the setting or individuals studied and provide systematic evidence for presenting the characteristics of perception diversity [29–31]. A working group (KR, JD, VS) was established to guide the development and implementation of this study. The working group (N = 3) consisted of one national and two international experts with experience in maternal and child health in the Lao PDR. The working group reviewed all research documents and tools and provided clarification during the data collection and analysis stages. They also provided the principal researcher (MW) with advice regarding the cultural appropriateness of research tools and conducting interviews in the local context.

Having satisfied herself through the literature review and in discussion with the working group that the introduction of community distribution of misoprostol was a potentially life-saving intervention, the principal researcher did not make a decision to support or oppose the proposed intervention per se, nor its suitability in the Lao context; rather she maintained a neutral position throughout the data collection phase. The principal researcher worked with an interpreter (VV), a medical doctor, and trained researcher, with significant experience in maternal and child health and stakeholder analysis. This person did not have an in-depth, prior knowledge of misoprostol use, and did not express support or opposition for the proposed intervention to the researcher or to the participants in the interviews.

A half-day training session was carried out with the researcher, interpreter and member of the working group prior to the data collection phase. Translation of the interview question guide was crosschecked by the working group members for linguistic accuracy. Interview questions were also reviewed for appropriateness to ensure they would facilitate a rich and open source of information from respondents in the local context.

### The study setting

The study setting was Vientiane and a northern province in the Lao PDR (the name of the province has been withheld to protect confidentiality and avoid deductive disclosure). The province is mountainous and geographically challenging, and with a high proportion of ethnic minorities and disadvantaged populations. The two areas were selected because Vientiane is the capital city of Lao PDR, where the majority of the key stakeholders were based. The province was selected because it has been identified by the Lao Society of Obstetrics and Gynaecology (LSO&G) as one of the most appropriate settings for a future pilot program of misoprostol to prevent PPH and has a high level of maternal mortality.

### Sample

Through initial discussions with the working group and local informants, and during the interview process, a list of key stakeholders were compiled and key informants identified using purposive sampling and snowballing. Selection criteria included informants’ professional knowledge and experience, as well as their influence and involvement in maternal and reproductive health policy-making, and willingness and availability to participate in the research. New key informants were added until no additional stakeholders were identified and saturation was reached. Following Buse et al. [24], the stakeholders were categorised as 1) government (departments and mass organisations), 2) health care managers and providers (hospital management, doctors, midwives, nurses and pharmacists), 3) non-government organisations (international organisations, international NGOs, bi-lateral organisations research institutions, professional organisations and the private sector), 4) civil society, and 5) community (women, family, community health workers, traditional birth attendants, community and religious leaders). In total, 35 stakeholder interviews were undertaken (see Table 1). While the LSO&G were identified as important stakeholders, they declined to be interviewed because they initiated this stakeholder analysis and felt that any information they provided would be biased.

**Table 1:** Stakeholder groups, subgroups and numbers interviewed.

Stakeholder group	Stakeholder subgroup	Number interviewed
International	International organisations	3
	International NGO	5
	Bilateral organisation	1
	International research institution	1
Government	Ministry of Health departments	6
Healthcare managers and providers—central level	Hospital management	1
	Heads of obstetrics and gynaecology departments and doctors	2
	Heads of Pharmacy departments	3
	Head obstetrics and gynaecology nurses	3
Healthcare managers and providers—provincial level	Hospital management	1
	Obstetrics and gynaecology doctor	1
	Head of Pharmacy department	1
	Head obstetrics and gynaecology nurse	1
Healthcare managers and providers—district level	Hospital director	1
	Obstetrics and gynaecology doctor	1
Healthcare providers—village level	Medical doctor	1
	Midwife	1
	Nurse	1
Private—central level	Medical doctor, private health centre	1
	**Total**	**35**

### Data collection

Semi-structured, in-depth individual interviews were undertaken using a semi-structured interview guide. On one occasion, two representatives chose to be interviewed together. One respondent answered via email as it was not possible to meet face to face. The semi-structured interview guide ensured specific topics were covered while also allowing the interviewees’ responses to determine the information collected and its relative importance [32]. Questions in the guide were a mix of open-ended and closed questions. Closed questions related to prior awareness, levels of support and distribution models. Open questions related to concerns, resources, barriers and actions, additional information required, alliances and leadership. One question also asked the respondents to categorise their position in terms of ‘strongly support’, ‘somewhat support’, ‘neutral’, ‘somewhat oppose’ or ‘strongly oppose’. Interviews were undertaken in a place of convenience for the interviewees, typically in their workplace and conducted in English or Lao depending on the preferred language of the respondent.

### Translation

The interpreter translated the data collection tools and the informed consent form into Lao. The interpreter simultaneously translated during interviews with Lao speaking stakeholders. Interviews were transcribed into English and checked against the Lao interview summaries. Almost inevitably however, in the interpretation process, editorial decisions were made and some nuances may have been lost. The interpreter also provided any required clarification for the data analysis stage and assisted in the preparation of the report and manuscript. The interpreter also assisted with the crucial tasks of scheduling the Ministry of Health (MoH) stakeholder appointments and associated administrative tasks, such as request letters.

### Data analysis

A stakeholder data analysis table was developed based on the Health Reform Tools Series, *‘Guidelines for conducting a stakeholder analysis’* [33], adjusted to ensure appropriateness and particular relevance to this project and its context. Data were also analyzed by counting frequencies of responses [29, 32]. This was not to add greater generalizability, generality or to make claims about causality of the conclusions but allowed checking of patterns and in some cases provided a more precise way of quantifying stakeholder views than the use of terms such as “many”, “several”, and a “few” [32, 34]. Incorporating frequencies in qualitative research can contribute to the internal generalisability of claims, within the setting of the individuals studied, and provide systematic evidence for presenting the characteristics of perception diversity [30]. Following Schmeer [33], we assessed each stakeholder’s level of influence based on their level of power and leadership role. Power was defined as the quantity of resources and ability to mobilize those resources for, or against, a particular policy. Leadership was defined as a willingness to initiate or lead an action for or against the policy [33]. Using these definitions, we analyzed information that was directly reported by stakeholders in the interviews, as well as indirect information gathered through other stakeholders, secondary information, and others’ perceptions. Where a discrepancy was found between the self-reported stakeholder’s position, and that perceived by others, this was checked against other available information from interviews and organizational policy position. We then divided the stakeholders into three groups: Group 1 –those who had leadership and high power (level 3); Group 2 –those who had leadership and medium power (level 2); Group 3 –those who did not have leadership but had high to medium power (level 2 or 3) [33].

Stakeholders were also divided by their level of knowledge around the use of misoprostol for prevention of PPH with level one being the lowest. A low level of knowledge (level 1) was based on the respondent not being aware that misoprostol could be used preventatively but were aware of its use to treat PPH or as an abortion drug. A moderate level of knowledge (level 2), was used to describe those who knew of misoprostol for prevention and treatment, and were aware of some of the concerns surrounding its use [33]. A high level of knowledge (level 3) referred to those who were aware of misoprostol’s different uses and the concerns surrounding its use. The information from these different analyses were tabulated (first by MW and subsequently checked by JD) and then compared and cross-referenced to produce a stakeholder position map [33]. This allowed for the analysis to be presented graphically, illustrating which actors supported or opposed community distribution of misoprostol and how important that position may be to the success of any subsequent pilot program [33]. A report summarizing the findings was translated into Lao and presented to key stakeholders in the MoH and the LSO&G.

### Ethics

The study was carried out after approval from the National Ethics Committee for Health Research, Ministry of Health of Lao PDR. Ethics approval was also given by the University of Queensland’s School of Population Health Committee. Information about the voluntary and confidential nature of the project was given verbally prior to the interviews. All respondents agreed to participate and steps were taken to ensure their confidentiality. Verbal consent was approved by the the University of Queensland’s School of Population Health Committee and the National Ethics Committee for Health Research, Ministry of Health of Lao PDR. Verbal consent was approved as the informants were personnel in national or international agencies, responsible for the health issues to be discussed. The information sheet and written informed consent form were available as a guide to obtaining verbal informed consent at both the beginning and end of interviews. A witness was present and verbal consent documented. The principal researcher and the working group discussed the potential conflict of individual or organisational respondents being identified in the final report and this paper and the effect this may have on them answering questions meaningfully. Following these discussions, it was decided to protect confidentiality to mitigate the potential of individuals not answering honestly if they thought they could be identified, as this was determined to represent a significant risk. Stakeholders were informed therefore that the identity of individuals, departments and organisations would be protected for confidentiality in published reports. We were however aware of the possibility of deductive disclosure, which occurs when the traits of individuals or groups are identifiable in research reports, and respondents were informed of such a possibility.

## Results

### Knowledge about misoprostol to prevent postpartum haemorrhage

All of the respondents included in this study reported knowing about misoprostol to treat PPH (n = 35), and 60 per cent (n = 21) were aware of misoprostol’s preventative use. At the provincial and district level hospitals however, hospital management were unaware of the use of misoprostol to prevent PPH. Similarly, nurses and midwives at the central, provincial, district and village levels were also not aware of the prophylactic use of misoprostol. Stakeholders were rated as having low, medium or high levels of knowledge about misoprostol. Almost all of the stakeholders from international organizations demonstrated high levels of knowledge as did several central level MoH stakeholders. Provincial and district levels had moderate to low levels of knowledge with community-based workers and nurses mainly demonstrating low levels of knowledge.

The majority of stakeholders, 68.6 per cent (n = 24), also said that before the introduction of misoprostol for preventing PPH in Lao PDR, they would like more information from international experiences in low-resource settings. The most commonly requested information across stakeholder groups was for experiences from other countries, information on any long-term effects and evidence for the Lao context. Table 2 shows the type of information the different stakeholder groups requested.

**Table 2:** Information needs identified by stakeholder subgroups on the community distribution of misoprostol.

Stakeholder groups	Information required to support misoprostol to prevent PPH	Example quotes to illustrate points
INGOs	Neighbour country related policy and practice and experience of synchronising oxytocin and misoprostol	“[we] need to be aware of what the neighbouring countries are doing, what is the practice around the region—how is misoprostol being managed in Thailand, Cambodia and Vietnam—does it make sense”
	Evidence for community distribution of misoprostol and distribution models	“Studies elsewhere have shown that the distribution of misoprostol in clean birthing kits (CBK) encouraged facility-based births because increased knowledge about PPH encouraged women to seek support of facility—information about misoprostol should be widely disseminated”
	Pilot/trial in Lao PDR	“a pilot program would be needed first with robust evaluation”
	Mapping of Lao health centres and villages including distances and travel times by vehicle, boat, and foot	“It is important to first map health centres and villages including distances and travel times by vehicle, boat, and foot as different distribution models will vary from area to area according to local conditions”
Ministry of Health	Pilot program /trial in Lao PDR	“there should be a trial first” “I would support wide distribution after a trial if conclusions were good”
	Lessons from other countries	“I know about the Bangladesh experience where there was a significant reduction to MMR—disseminating lessons from other countries is important”
	Recommendations from this stakeholder analysis	“the results of this stakeholder analysis should be disseminated”
Central Hospital Management and Doctors	Guidelines	“Guidelines and policy are needed, including complications, how to give safely to reduce the maternal mortality rates from PPH” “Guidelines should be disseminated to doctors and healthcare workers” “Without guidelines some doctors may be concerned regarding safety and uterine rupture if uncontrolled”
Central and Provincial Hospital Pharmacy	Long term side-effects Lessons from other countries	“we need to know more about lessons learned from other countries, complications and side effects”
	Trial/pilot program in Lao PDR Guidelines	“needs to be information from a trial”
	Guidelines	“there needs to be guidelines”
Central and Provincial Hospital Nurses	Training	“training is needed”
	Trial/pilot program in Lao PDR	“I am concerned about evidence in Laos—no evidence for use in communities and a trial is needed”
	Lessons from other countries	“[I am] interested to know the experiences of other countries”
Provincial Hospital Management and Doctors	Lessons from other countries	“We need information from implementation in other countries. Then can think of how best to in Laos”
District Hospital Management and Doctors	None raised	
Village Health Centre Doctor, Nurse and Midwife	None raised	

### Support for community distribution of misoprostol

Most stakeholders were supportive of misoprostol, although their influence differed greatly. Some of the major international players who were in positions of influence were somewhat ambivalent, yet none of the stakeholders showed a complete lack of support. Many of the respondents in the Lao healthcare system, especially those with higher levels of knowledge, were very supportive as one female health professional at the central level asked “why let women die?” Further, key stakeholders did not identify any stakeholders who they felt would actively oppose misoprostol to prevent PPH. Several stakeholders believed there would be some disagreement in the background amongst medical colleagues and in the general community but felt the situation could be managed appropriately with information and education. A respondent stated:

“*There will be no problems (with misoprostol)…the contraceptive pill has been accepted by the community. Look, there will be a few rumours, just like there was with condoms encouraging sex. People might say that misoprostol will encourage abortion, but information and time and the myths will disappear.*”

Most people did not think there would be much opposition to the introduction of misoprostol for the prevention of PPH, as one doctor explained:

“*I do not think there will be opposition if misoprostol is used for prevention of PPH but I don’t think people will agree if it is also available for medical abortion*”

The overwhelming majority of participants interviewed noted that the women who would benefit most from community distribution of misoprostol would be those in rural areas with limited access to skilled birth attendants. Despite this, while most were supportive of distribution of misoprostol by trained healthcare staff, there was opposition to misoprostol distribution by unskilled birth attendants such as: village health volunteers, family members, or self-administration by women themselves (see Table 3). There were also concerns about the current level of training of village health volunteers and their lack of recognition in the formal public health system, as well as their capacity given their already heavy workload. The quotes below reflect common sentiments about the capacity of the village health volunteers and are reflective of concerns at both central and provincial levels:

“*Many VHV are semi-illiterate or illiterate and they are the least-trained health cadre and not formally recognised as part of the health system.*”

“*The problem is that VHV are often busy with their other local authority roles such as village chief and we keep asking them to do more and more things for TB, malaria, immunisation, sanitation and hygiene.*”

**Table 3:** Stakeholder support for cadres of people for the community distribution of misoprostol.

Cadre of people for community distribution of misoprostol	Number (and %) of stakeholders who agree to the cadre
Trained health care staff	30 (85.7%)
Semi-skilled birth attendants	25 (71.4%)
Unskilled birth attendants	14 (40%)
Women themselves	9 (25.7%)

Nevertheless, while the majority of stakeholders supported distribution of misoprostol in the community by trained healthcare staff and semi-skilled birth attendants, there are however, significant shortages of trained healthcare staff and semi-skilled birth attendants in the Lao PDR. Many stakeholders recognized this, but felt that distribution models would need to be adapted to local contexts in the Lao PDR. The responses of two stakeholders interviewed help to illustrate this:

“*Misoprostol is a very safe drug…but I would not condone any woman carrying out an unassisted abortion…I fully support the community distribution of misoprostol in a maternal health environment with pregnant women who intend to give birth. Misoprostol can be given out or in some areas it may be better to include it in clean birthing kits.*”

“*This should be considered on a case by case basis. Different areas will need things done in different ways.*”

### Concerns related to community distribution of misoprostol

Many of the concerns associated with the introduction of misoprostol related to its potential misuse and concerns about the safety of the drug, especially in the hands of low-skilled community health workers and families themselves. All respondents agreed they would support the community distribution of misoprostol only with appropriate training and ongoing support for the people distributing the medication and the community. Another common theme was that the community distribution of misoprostol should only be through the public system with appropriate regulations and monitoring to ensure drug quality and to address the potential for misuse. Lack of technical resources and potential issues in the supply and distribution system however, were also frequently mentioned. Stock outages and delays in essential medicines in the public system for example, were reported as common. While private pharmacies were reported to have a more consistent supply of medications, concerns were raised about weak regulation and the practice of private pharmacies selling medical products without a prescription. Misoprostol and Chinese brands were described as already being available in private pharmacies and sold without proper counselling. These quotes from health staff at the central and provincial level help to exemplify these concerns:

“*We don’t have data about exactly how it [misoprostol] is being used but we know it is [being used] because doctors are dealing with the women who present at the hospital with complications*”

“*People are taking Chinese medicine then present at the hospital with the tablets—they get the Chinese tablets from the Chinese clinic*”

“*There needs to be controls otherwise sellers will advertise and people will misuse [the drug]*”

According to one pharmacist:

“*In urban areas—misoprostol is known as the ‘drug for letting the blood out’–that’s what the women ask for when they miss their menstruation*”

Another concern regarding community distribution of misoprostol that emerged from interview data was whether prioritizing misoprostol through policies or programs could undermine efforts to improve access to safe delivery services and active management of the third stage of labour. Some participants noted that access to misoprostol to prevent PPH may compound current low utilization of facility-based delivery. The general consensus among those interviewed however, was that misoprostol was an important option in reducing maternal mortality that to date, had been given limited attention at the central level.

Many of the interviewees noted that support and advocacy by obstetricians and gynaecologists would be critical in getting misoprostol included in policy and clinical management protocols for the prevention of PPH. The capacity of the Lao MoH to fund a pilot program of community distribution of misoprostol was also questioned, and many thought that any pilot program would require external donor funding, raising questions of sustainability and equity in access. Training in its use, specialist support, and close supervision were also consistently mentioned, reflecting other concerns about the capacity of lay community members to use misoprostol safely and appropriately. Strengthening the capacity of village health workers was also identified as key if community distribution was to be introduced. Informants also mentioned that for many rural communities, PPH is seen as an inherent and unavoidable risk of giving birth and community education would be vital in any effective community distribution of misoprostol strategy. Most of the international stakeholders also suggested that effective monitoring across all levels of the health system would be an important issue for the implementation of any future introduction of misoprostol. International stakeholders also mentioned the importance of awareness raising and developing demand at the community level.

### Stakeholder influence

The stakeholder analysis identified the key stakeholders who can influence policy development for the community distribution of misoprostol to prevent PPH in Lao PDR. These included the MoH and its departments, and several NGOs including: international organisations, a bilateral organisation, an international NGO, and an international research institute. The MoH are the key decision-makers, yet the influence of the opinion leaders is also significant and can translate directly into technical advice and support for the MoH. Within the MoH, the LSO&G were identified to be particularly influential. All MoH stakeholders agreed they would take the advice of the LSO&G and would be open to considering proposals for a pilot program. This finding suggests that amongst the key MoH stakeholders there is significant cohesion and support for the LSO&G and for continued exploration of the potential use of misoprostol to prevent PPH. All stakeholders reported willingness to work collaboratively with all other relevant stakeholders and develop strategic relationships as required. District-level stakeholders were identified as key implementers and vital in any pilot program, yet district staff had very limited understanding of the preventative use of misoprostol. The LSO&G has also engaged with international organisations, relevant international academics and their associated institutions regarding the community distribution of misoprostol however the international actors included in this study expressed somewhat ambivalent support. Such government and NGO political and technical support is important in terms of funding, but also in ensuring any intervention is rigorously monitored and evaluated.

Stakeholder power—that is, the influence a stakeholder has over the policy and the degree they can help achieve or block the desired change [33], was established by reviewing the specific resources of each stakeholder. A stakeholder position map was then developed based on the stakeholder’s power and their support or opposition [33]. In our original questionnaire, we asked respondents to identify as a “supporter”, “moderate supporter”, “neutral”, “moderate opponent” or “opponent” which we subsequently combined into “supporter”, “neutral”, or “opposed” (Table 4). As Table 4 illustrates, almost all respondents could be classified as supporters (n = 31) and there were no opponents. One person in the MoH with a high level of power, one international organization, and one INGO, all with high levels of power, all declared neutral positions. Based on Table 3 and the knowledge needs, these individuals and organizations may need to be provided with more evidence from similar contexts to support community distribution of misoprostol but are unlikely to oppose a pilot program. The other stakeholder who reported a neutral position (Central Hospital Nurse) has a low level of power and is very unlikely to be able to effectively oppose any pilot program or introduction of community distribution of misoprostol. This is not to say, however, that this person’s needs and concerns should not be addressed.

**Table 4:** Stakeholder position map.

	Low	Medium	High
Position			
**Supporter**	Private Doctor (n = 1) Central Hospital Nurse (n = 2) Provincial Hospital Nurse (n = 1) Village Health Centre Doctor (n = 1) Village Health Centre Manager (n = 1) Village Health Centre Nurse (n = 1)	INGO (n = 4) Central Hospital Manager (n = 1) Provincial Hospital Manager (n = 1) District Hospital Manager (n = 1) Central Hospital Doctor (n = 2) Provincial Hospital Doctor (n = 1) District Hospital Doctor (n = 1) Central Hospital Pharmacy (n = 3) Provincial Hospital Pharmacy (n = 1)	Ministry of Health (n = 5) INGO (n = 1) Bilateral Organisation (n = 1)
**Neutral**	Central Hospital Nurse (n = 1)	None	Ministry of Health (n = 1) International Organisation (n = 2)
**Opposed**	None	None	None

When asked who should lead the introduction of misoprostol to prevent PPH, almost all felt it should be the MoH. Two participants from INGOs felt the MOH should take the lead in partnership with an international organization. The LSO&G and O&G doctors were also identified as an important leadership group.

## Discussion

The LSIS shows that Lao PDR has made progress in reducing maternal mortality [12]. Much of the improvement is likely to be due to the reduced fertility rate rather than improved access to appropriate and effective maternal healthcare including essential obstetric care [12, 13]. Further, maternal mortality is likely to be underreported due to problems with accurate reporting of deaths at the community level especially in remote areas. In addition, while the MMR has decreased significantly in Lao PDR, it is still the highest in the region. Furthermore, in country- inequalities in access to emergency obstetric care and maternal health outcomes are evident [12]. Addressing inequity in maternal mortality among different geographical, socioeconomic and cultural populations is an important part of Lao PDR’s contemporary health strategy.

The recognition that there is a social gradient in health is receiving increasing interest in public health and often it is the people most in need that are hardest to reach. While important gains have been made in reducing maternal mortality in the Lao PDR, progress has been achieved by a disproportionate improvement in the burden of maternal mortality in urban populations and continued inequitable progress is unlikely to lead to sustainable maternal health outcomes. As in neighboring Vietnam, economic factors are only one generator of inequity in the Lao PDR [35]. Addressing inequity in maternal health outcomes due to the social and economic gradient in health may require tailored interventions for different ethno-linguistic, geographical or otherwise differentiated populations [36]. There is compelling evidence to suggest that the the provision of misoprostol to prevent PPH—a leading cause of maternal mortality—is an effective strategy when implemented alongside efforts to increase access to essential obstetric care and facility-based birthing [2–4, 9, 22]. Future efforts should also include addressing the other socio-economic determinants of health. Important in this, is disaggregating indicators on reproductive and maternal health and access to healthcare based social stratifiers such as wealth quintiles, gender, urban/rural residence, ethnicity and educational status, in order to monitor progress towards more equitable outcomes [35, 37].

While misoprostol is included in the Lao National Essential Medicines List for a variety of indications at the central and provincial levels, it is not included for use at district and village levels and has not been prioritized as a tool for reducing maternal mortality from PPH. Given this, it is not surprising that there was very limited knowledge of misoprostol to prevent PPH at the provincial, district and village levels, yet it is at these levels where most births without a trained health professional are likely to occur [12, 13, 21]. Of concern, is that maternal death as a result of PPH was reported as so common as to be normalised in many rural areas, suggesting the need for further education at the village level and increasing demand for services and preventative interventions.

The introduction of community distribution of misoprostol has been controversial in the Lao PDR and the legitimate stakeholder concerns provide some answers as to why a relatively low-cost, easy to administer and potentially life-saving drug has not been prioritised in not only reducing maternal mortality but also reducing urban and rural disparities in maternal mortality. Despite the concerns, the consensus among those interviewed in this study was that misoprostol may be an important option in reducing maternal mortality due to PPH. One common concern among international stakeholders, as well as the MoH and some senior central hospital doctors, was that community distribution of misoprostol to prevent PPH could endanger the promotion of facility-birthing and commitment to improving access to skilled birth attendants, especially as many women and families continue to prefer to deliver at home rather than in public facilities [21]. Evidence from Nepal, Afghanistan, and Zambia suggests that misoprostol can be used without compromising rises in facility-based delivery [4, 9]. Nevertheless, to assuage fears, community-based misoprostol interventions should be implemented alongside community-based educational campaigns promoting facility delivery where possible [2]. Concurrently, further effort is needed to address the multiple barriers that prevent women in the Lao PDR from delivering in a heath facility [13, 21].

Other safety concerns related to home-based distribution by relatively low-skilled providers were consistently raised by national and international stakeholders. Experience from other low-resource settings however, suggests community or home-based distribution can be achieved safely and with high rates of coverage in the target population [2, 4, 5, 22, 23, 38]. Further, given the absence of other feasible management options at the community level for prevention of PPH during home deliveries, and the numerous barriers to women in the Lao PDR reaching a secondary or tertiary level facility which can provide essential obstetric care [13, 21, 39], there is a strong argument for preventative measures at the community level. Without any intervention, a woman can die from PPH in the immediate post delivery period [40] and even if she survives, severe anaemia and other morbidities may compromise overall health, productivity and subsequent birth outcomes into the future [41]. Given this, and that in the rural settings where misoprostol is needed there is a lack of trained healthcare providers to administer misoprostol at the time of delivery, further advocacy and dissemination of lessons learned from other similar settings is needed. In addition, any pilot program should be accompanied by collection of appropriate data on health outcomes, coverage, and safety [11].

Many of the concerns mentioned by a range of stakeholders interviewed in this study related to the capacity of village health volunteers and community members to safely manage the distribution and administration of misoprostol. This study indicated that stakeholders at the district and community levels would be critical if a women and untrained health workers administered model were to be considered. It is these very stakeholders who appear the have the least amount of knowledge which reflects the need for an effective education campaign as a critical component for successful community distribution of misoprostol [2]. In addition, given pharmacies are often the first place people seek treatment in the Lao PDR [13, 42], designing and implementing appropriate training for community-based drug sellers is also important in helping to prevent incorrect usage and potential negative outcomes [2]. In addition, the piloting of community distribution of misoprostol for the prevention of PPH and any subsequent scale-up would need careful monitoring and evaluation to develop an evidence base and ensure that the scope of any intervention extended only to the extent warranted by the evidence base [2]. Other issues raised related to competing priorities and a general lack of financial and technical resources.

Four stakeholders from international organisations, all identified as powerful decision-makers and in positions to influence policy, were ambivalent about the use of community or home-based distribution of misoprostol despite the *2012 World Health Organization guidelines for the prevention and management of PPH* recommending oral administration of misoprostol by community health workers [11]. This is significant because these stakeholders all have a high degree of influence over the development of health related policy in the Lao PDR and funding for any pilot program is almost certainly going to need support from the international community. While one stakeholder declared misoprostol as a “*political hot potato”*, although generally ambivalent, these international stakeholders did suggest that while cautious, they would support changes in current policy if requested. On the one hand, waiting for the national authorities to take the lead in conceptualising and driving appropriate policies is desirable. On the other hand, the lack of domestic funding and the often weak leadership capacity of state agencies leaves the LSO&G, who are advocates for misoprostol and would take a lead in piloting community distribution, in a challenging position in taking forward the agenda of improved and more equitable maternal health outcomes. While understandably cautious, the international community could provide the LSO&G and the government more support and technical assistance in developing the capacity of lower-level providers and women who face the highest risk of maternal mortality and morbidities and their families, to improve maternal outcomes.

As with all studies, this study has some limitations. Firstly, the purposive sampling strategy and limiting the data collection to two geographical areas, means the sample is not representative. The purpose of this research however, was not to generalise the findings. Stakeholders involved small groups of organisations and individuals who were known to each other. While confidentiality was assured, the political nature of some of the questions may have influenced some interviewees to temper their responses. The interviewer’s and interpreter’s personal knowledge and attitudes may also have biased the interpretations. However, the researcher declared a neutral position at the beginning of each interview highlighting the project was a stakeholder analysis with the aim to investigate and document the different stakeholder perspectives rather than trying to prove or disprove a hypothesis. Finally, as many of the interviews relied on translation, inevitably, some nuances may have been lost.

## Conclusion

Despite gains in maternal health outcomes, the maternal mortality ratio remains unacceptably high in the Lao PDR and disparities by location and ethnicity are evident. Efforts to ensure universal access to skilled birth attendants and essential obstetric care are long-term endeavours that require substantial health system strengthening. While not wanting to sidestep the important issue of providing appropriate and effective essential obstetric care to every woman, community distribution of misoprostol has the potential to save lives by reducing PPH. Access to easy-to-use oxytocic drugs is a matter of health equity and for home births misoprostol is currently the only feasible solution for effective prevention. This is not to undermine the need to continue efforts toward reducing inequities in delivery by skilled birth attendants through demand-enhancing and supply-side cost-effective measures. Failure to do so will ultimately allow inequalities to persist and community distribution of misoprostol must be implemented alongside advocacy and increased referral and uptake of antenatal care and facility-based birthing as well as interventions that address the social determinants of maternal mortality.

Operational research is also needed to demonstrate the potential reductions in maternal morbidity and mortality associated with community level administration of misoprostol and allay stakeholders’ valid concerns surrounding misoprostol use at the community level. Ultimately, the extent to which misoprostol is utilized in a particular setting will depend on many factors, including demand for the drug from providers and individuals, and the socio-cultural context in which the drug is introduced. Examining and documenting the legitimate concerns among stakeholders involved in maternal and reproductive health policy and services can provide insight on the reasons why certain interventions are not implemented. It can also help in developing a communication plan and call to action around a specific issue. Finally, given the transient nature of policy-making, a detailed stakeholder analysis helps to track shifting interests so that policy strategies can be modified to achieve more sustainable policy changes.

## Supporting Information

S1 Question guide (DOCX)
