# Supplementary material for: Stakeholder Analysis of Community Distribution of Misoprostol in Lao PDR: A Qualitative Study
Source: PLoS One. 2016 Sep 15;11(9):e0162154. doi: 10.1371/journal.pone.0162154 (PMC5025235; doi:10.1371/journal.pone.0162154)
Supplement: S1 Question guide — (DOCX) [file pone.0162154.s001.docx]

**Supporting information: Question guide**

**Community distribution of misoprostol for the prevention of postpartum hemorrhage: a stakeholder analysis Lao PDR**

Date:

ID #:

1. What do you think are the priorities for maternal health (MH) in Lao?

2. What are your organisation’s main activities around MH in Lao?

3. Have you heard about misoprostol for the **prevention** of postpartum hemorrhage (PPH)?

4. If so, where did you hear about the community distribution of misoprostol to prevent PPH?

5. Is your organisation doing any work with misoprostol and what are the activities?

6. Are you aware of any concerns around the use of misoprostol in Lao?

7. Which of these categories best describes your organisations’ opinion on misoprostol to prevent PPH for women who do not have access to skilled birth attendants and facility based care?

a) I strongly support it

b) I somewhat support it

c) I do not support nor oppose it

d) I somewhat oppose it

e) I strongly oppose it

Why?

8. Which of the following community distribution models for misoprostol and the prevention of PPH do you support in Lao?

a) Distribution by trained health care staff

b) Distribution by semi-skilled birth attendants (such as community health workers)

c) Distribution by unskilled birth attendants (such as village health volunteers and family/community)

d) Distribution to pregnant women themselves for self-administration after delivery of the baby

Why?

9. If you support misoprostol for the prevention of PPH, how would you demonstrate this support?

- Would you have any resources to dedicate to supporting such a program?

- Would you cooperate with any other persons or organisations?

- Would you take the initiative in supporting misoprostol, or would you wait for others to do so?

- How quickly would you be able to mobilise your support?

10. If you do not support misoprostol for PPH, how would you demonstrate this opposition?

11. What do you think the main barriers to promoting and implementing the community distribution of misoprostol for the prevention of PPH in Lao?

- Research (clinical and operational)

- Policy / advocacy

- Project / program implementation

- Community (socio-cultural) acceptance

12. What actions could be taken to overcome these barriers:

- By policy makers

- By health providers

- By international organisations

- Are there any other key actors (community / women’s groups)?

I would now like to ask you a few specific questions about your opinion regarding others opinion of misoprostol to prevent PPH.

13. What other organisations, or departments, or persons do you think would support misoprostol to prevent PPH in Lao?

14. Which of these supporters would take the initiative to actively support such a program?

15. Which of these supporters would work together to demonstrate their support for such a program?

16. Are you aware of any organisations or departments who are opposed to misoprostol for PPH?

17. Do you know why they are opposed?

18. Is there any further information or research around misoprostol for the prevention of PPH that you would like to know?
